# Os LncRNAs Estão Envolvidos no Processo de Aterosclerose em Diversos Níveis

**DOI:** 10.36660/abc.20201383

**Published:** 2022-06-06

**Authors:** Shiyi Liang, Weicheng Xv, Chijian Li, Yuxiang Huang, Ge Qian, Yuxiang Yan, Hequn Zou, Yongqiang Li

**Affiliations:** 1 The Third Affiliated Hospital of Southern Medical University Department of Nephrology Guangzhou China The Third Affiliated Hospital of Southern Medical University – Department of Nephrology, Guangzhou – China; 2 Capital Medical University Department of Epidemiology and Biostatistics Beijing China Capital Medical University – Department of Epidemiology and Biostatistics, Beijing – China; 3 South China Hospital of Shenzhen University Department of Nephrology Beijing China South China Hospital of Shenzhen University, Department of Nephrology, Beijing – China; 4 The Third Affiliated Hospital of Southern Medical University General Practice Department Guangzhou China The Third Affiliated Hospital of Southern Medical University – General Practice Department, Guangzhou – China

**Keywords:** LncRNAs, Enzimas, Inibidores Enzimáticos, Inibidores, Aterosclerose, Doenças Cardiovasculares, Células Endoteliais, Lipoproteínas VLDL, Interferência de RNA

## Abstract

A aterosclerose é a causa mais comum de doença cardiovascular em todo o mundo, ela está associada a uma alta incidência de eventos clínicos. O acúmulo de evidências elucidou que os RNAs longos não codificantes (LncRNAs) são uma nova classe de transcritos com papéis críticos nos processos fisiopatológicos da aterosclerose. Nesta revisão, resumimos o progresso recente dos LncRNAs no desenvolvimento da aterosclerose. Descrevemos principalmente os diversos mecanismos regulatórios dos LncRNAs nos níveis transcricionais e pós-transcricionais. Este estudo pode fornecer informações úteis sobre os LncRNAs como alvos terapêuticos ou biomarcadores para o tratamento da aterosclerose.

## Introdução

**Figura 1:**
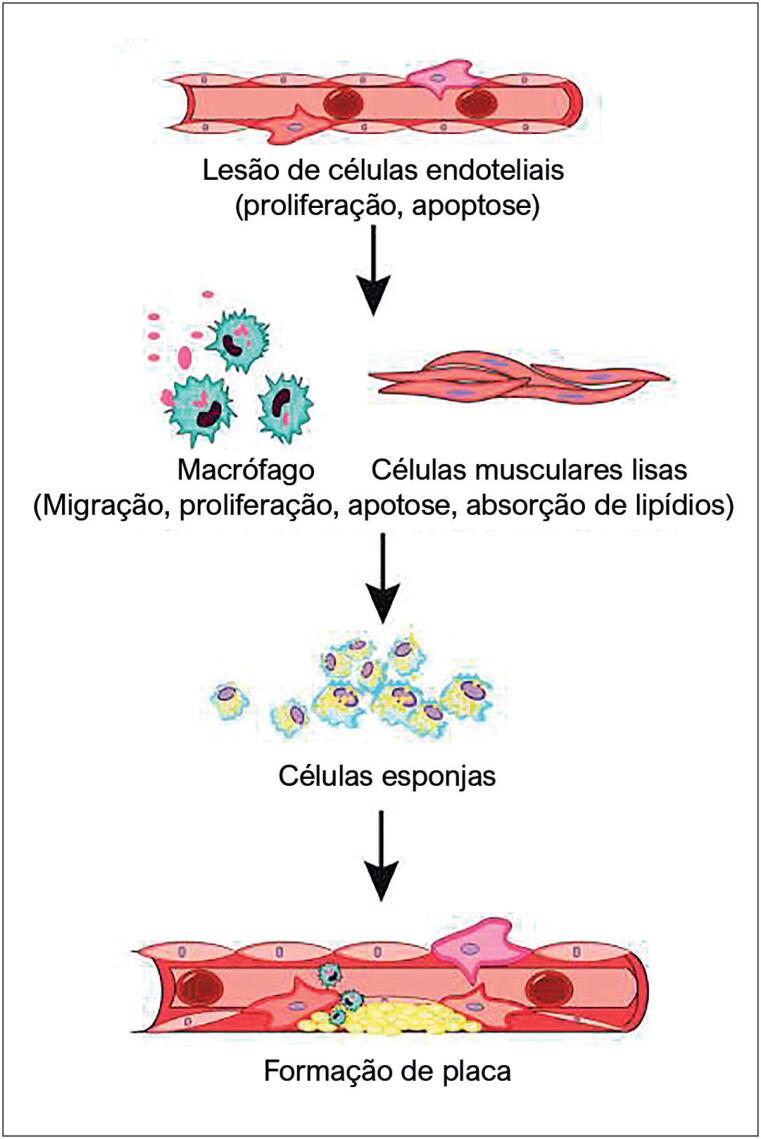
A patogênese da aterosclerose.

As doenças cardiovasculares (DCVs) são consideradas um problema de saúde global, responsável por 17,9 milhões de mortes todos os anos.^1^ A aterosclerose (AS), a principal impulsionadora das DCV em todo o mundo, é um processo inflamatório crônico conduzido por lipídios com disfunção endotelial, formação de células espumosas e acúmulo final de placa.^2^ Este processo é acompanhado pela proliferação de células, apoptose e liberação de fatores pró-inflamatórios.^3^ (Figura 1) Eles podem desencadear a ruptura da placa e a formação de trombose, levando a eventos clínicos agudos, como acidente vascular cerebral e síndrome coronariana aguda.^4^

No genoma de mamífero, os RNAs codificantes de proteínas são apenas <3%.^5^ Essa fração do gene codificador torna, portanto, difícil explicar o complexo mecanismo regulatório do organismo. Nos últimos anos, estudos têm revelado o importante papel dos RNAs não codificantes de proteínas nos processos fisiopatológicos de várias doenças.^6–7^ De acordo com o comprimento, os RNAs não codificantes (ncRNAs) podem ser divididos em RNAs não codificantes longos (lncRNA, >200 nucleotídeos) e RNAs não codificantes pequenos (<200 nucleotídeos, como miRNAs, piRNAs e siRNAs).^8^ Em muitas pesquisas, algumas funções regulatórias e efeitos biológicos de pequenos ncRNAs foram demonstrados.^9–11^ A função de muitos LncRNAs é desconhecida, mas um número crescente de LncRNAs foi caracterizado.

A biossíntese do lncRNA é semelhante à do mRNA. Os LncRNAs são transcritos pela RNA polimerase II, mas não possuem fases de leitura abertas e estão em uma expressão mais baixa do que os genes codificantes de proteínas.^8^ Os LncRNAs estão localizados principalmente no núcleo e no citoplasma.^12^ No citoplasma, os LncRNAs podem se ligar aos ribossomos^13^ ou originar-se do genoma mitocondrial.^14^ Os primeiros relatórios mostram que muitos LncRNAs não podem codificar proteínas porque não possuem fases de leitura aberta (ORFs) ou contêm poucas ORFs. Mas evidências emergentes sugerem que alguns LncRNAs contêm pequenas ORFs que codificam pequenas proteínas ou micropeptídeos, que são considerados reguladores-chave em vários processos biológicos.^8,15,16^ Estudos demonstram que os LncRNAs desempenham papéis críticos na função das células endoteliais e do músculo liso vascular (VSMC), na ativação de macrófagos, no metabolismo lipídico e a resposta inflamatória.^17,18^ Nesta revisão, discutimos principalmente a regulação dos LncRNAs que estão envolvidos no processo fisiopatológico da aterosclerose em níveis transcricionais e pós-transcricionais.

A patogênese da aterosclerose é acompanhada por disfunção celular, como proliferação, apoptose e migração. O resultado é a formação de células espumosas e o acúmulo de placas.

### As classificações e mecanismo regulatório de LncRNAs

De acordo com a correlação entre a localização genômica e genes codificadores de proteínas, os LncRNAs podem ser divididos em (1) LncRNAs intergênicos (LincRNAs) que expressam genes codificadores de proteínas como uma unidade independente. (2) LncRNAs intrônicos que derivam dos íntrons de genes codificadores de proteínas. (3) LncRNAs anti-senso transcritos da direção oposta dos genes que codificam proteínas. (4) LncRNAs senso que se sobrepõem aos exons de genes que codificam proteínas na mesma fita. (5) potenciadores que se originam no potenciador de genes que codificam proteínas. (6) LncRNAs bidirecionais que são transcritos a partir dos promotores bidirecionais divergentes.^19,20^ Os critérios de classificação também incluem as várias funções na regulação do gene local: cis- (regulação da expressão de genes proximais) e trans- (regulação da expressão de genes distantes).^21^ Além disso, os transcritos de LncRNAs também podem ser categorizados em lineares ou circulares.^22^

O mecanismo de funcionamento dos LncRNAs não foi completamente elucidado, mas pode ser classificado aproximadamente em vários grupos: 1. a regulação da transcrição está incorporada na interferência da transcrição, remodelação da cromatina e promoção da transcrição; 2. níveis pós-transcricionais se manifestam no controle da tradução da regulação do splicing de mRNAs e até mesmo como esponjas para miRNAs; 3. Outros contêm localização de proteínas, replicação de telômeros e interferência de RNA etc. Além disso, seus mecanismos de direcionamento para regular a expressão de genes são resumidos como o seguinte: sinais, iscas, guias e estruturas.^22,23^

### Regulação transcricional

Os LncRNAs podem exercer sua regulação transcricional por meio de mecanismos de ação cis e ação trans. (Tabela 1). Os LncRNAs regulam a expressão de genes vizinhos em cis via interferência transcricional ou remodelação da cromatina.^24^ Os LncRNAs de ação trans podem interagir com RNA polimerases e fatores de alongamento da transcrição ou servir como um arcabouço para complexos de modificação da cromatina para regular os genes distantes.^24,25^

**Tabela 1:** O papel dos lncRNAs no processo patológico da aterosclerose

	LncRNAs	Mecanismo	Efeito		Referências
Função celular			Proliferação	Apoptose	
Células Endoteliais (ECs)	MALAT1	MALAT1-Pc2 (CBX4)-E2F1	+		38
	GAS5	GAS5 - ceRNA (miR-21)	−	+	75
	HOTTIP	TNF-α/PDGFBB-HOTTIP-β-catenina	+		47
	MALAT1	ceRNA (miR-22-3p)		−	60
	TUG1	ceRNA (miR-26a)		+	71
Macrófagos, Células musculares lisas	ANRIL	Ligam com CBX7 e SUZ12	+	−	32
	NEAT1	NEAT1-WDR5-SM-genes específicos	+		44
	LincRNA-p21	LincRNA-p21-MDM2/ p300-p53	+	−	45
	HAS2	remodela estructura da cromatina	+		49,50
	RP11-714G18.1	regula positivamente a expressão de LRP2BP		−	53
	H19	ceRNA (miR-148b)	+	−	66
	MIAT	ceRNA (miR-181b)	+	−	69

	LncRNAs	Mecanismo	Efeito	Referências
Acúmulo de Lípidios	MALAT1	captação de lipídios MALAT1-CD36	+	45
	NEAT1	captação de lipídios NEAT1-CD36	−	41
	MeXis	LXR-MeXis-Abca1	−	46
	H19	ceRNA (miR-130b)	−	65
	TUG1	ceRNA (miR-133a)	+	72

	LncRNAs	Mecanismo	Efeito	Referências
Resposta inflamatória	ANRIL	TNF-α/NF-kB-ANRIL/YY1-IL6/8	+	34
	MALAT1	MALAT1-p65/p50-TNF-α e IL-6	−	40
	MALAT1	ceRNA (miR-503 or miR-155)	−	61,62
	H19	ceRNA (miR-130b)	−	
	NEAT1	ceRNA (miR-342-3p)	+	70
	TUG1	ceRNA (miR-133a)	+	72

(+) representa prompt ou aumento, e

(−) representa prevenir ou diminuir.

O estudo Wellcome Trust Case Control Consortium (WTCCC) e os estudos de associação do genoma completo descobriram que uma região no cromossomo 9p21 (Chr9p21) estava fortemente associada à doença arterial coronariana.^26^ A região é adjacente a um LincRNA denominado RNA não codificador anti-senso no lócus INK4 (ANRIL, também conhecido como CDKN2BAS).^27^ Holdt LM et al.,^28^ revelaram que a expressão de ANRIL estava correlacionada com a gravidade da aterosclerose por afetar a transcrição dos mRNAs, e o ANRIL também foi detectado em placas ateroscleróticas em seu estudo.^28^

Dois genes codificadores de proteínas, inibidores de quinase dependentes de ciclina (CDKN2A, CDKN2B) e a estrutura de leitura alternativa (ARF) no cromossomo 9p21, estão ligados a ANRIL inextricavelmente, os quais são supressores de tumor.^27^ O complexo polycomb repressivo-1 (PRC-1) e o complexo polycomb repressivo-2 (PRC-2) são dois tipos de proteínas de grupo polycomb envolvidas na manutenção do estado da cromatina.^29^ Suas subunidades CBX7 e SUZ12 se ligam ao ANRIL separadamente para silenciar o lócus CDKN2A/B através da trimetilação de H3 lisina^27^ (K27H3).^30,31^ Ainda, a repressão de CDKN2A/B pode estar relacionada à proliferação celular e apoptose no processo de aterosclerose.^32^

Holdt et al.,^28^ descobriram que ANRIL estava em posição de exercer uma função reguladora na expressão de genes distantes em trans. O elemento Alu, que marca o promotor dos genes trans-regulados de ANRIL, é decisivo para a trans-regulação linear de ANRIL. As proteínas PcG, desencadeadas pela ligação com ANRIL, eram altamente abundantes a jusante dos motivos Alu.^33^ O recrutamento de proteínas PcG poderia regular a expressão dos genes alvo (TSC22D3, COL3A1) e atenuar funções pró-aterogênicas mediadas por ANRIL, como células adesão, proliferação e apoptose.^3,33^ Além disso, ANRIL desempenha um papel fundamental nos processos inflamatórios através da via TNF-α/NF-kB-ANRIL/YY1-IL6/8. As proteínas associadas a PRC Yin Yang 1(YY1), um fator de transcrição, formam um complexo funcional com ANRIL.^33^ O complexo ANRIL-YYI se liga aos loci promotores de IL6/8 e estimula seu recrutamento na sinalização de TNF-α/NF-κB, levando à inflamação vascular.^34^

MALAT1, localizado no cromossomo 11q13, é descrito pela primeira vez como lncRNA associado a metástases de tumores de pulmão.^35^ A expressão de MALAT1 é regulada para baixo em placas ateroscleróticas em comparação com artérias não ateroscleróticas.^36^ Michalik et al.,^37^ descobriram que o silenciamento de MALAT1 inibiu a mudança de um estado promigratório para um proliferativo das células endoteliais, resultando na redução do crescimento de vasos.^37^ E MALAT1 também atua como um andaime molecular para interagir com o Polycomb 2 não metilado (Pc2); a expressão de Pc2 promove a SUMOilação de E2F1 e regula modificações de histonas para aumentar a proliferação celular.^38^

Em um experimento de controle, Gast et al.,39 observaram que os níveis séricos de TNF, IL-6 e IFN-γ estavam aumentados nos camundongos ApoE -/- deficientes em MALAT1, causando disfunção imunológica e aterosclerose agravada.^39^ MALAT1 pode estar envolvido na resposta LPS inflamatória induzida via sinalização LPS/TLR4/NF-κB. MALAT1 interage com as subunidades p65/p50 de NF-κB, inibindo a ligação de p65/p50 a promotores alvo, como TNF-α e IL-6, atenuando uma inflamação excessiva.^40^

No metabolismo lipídico, MALAT1 pode ser regulado positivamente em macrófagos durante a estimulação ox-LDL.^41^ CD36, um receptor eliminador de classe B, é necessário para a absorção de lipídios de ox-LDL.^42^ A superexpressão de MALAT1 induz o recrutamento de β-catenina no promotor CD36 para aumentar a transcrição de CD36, promovendo a captação de lipídios em macrófagos e acelerando a formação de células espumosas em placas ateroscleróticas.^41^

NEAT1, um transcrito adjacente de MALAT1, pode aumentar a formação de paraspeckles em oxLDL-macrófago induzido, que suprime a captação de lipídios ligando-se ao mRNA de CD36 para inibir a expressão de CD36 e estimula a resposta inflamatória por meio da fosforilação de p65 para promover a secreção de TNFα.^43^ Além disso, ASI et al.,^44^ descobriram que a expressão de NEAT1 foi regulada positivamente em células do músculo liso vascular (VSMCs) após lesão vascular in vivo e in vitro, levando a um estado de cromatina inativa em genes específicos de SM por meio da ligação com o modificador de cromatina WDR5. A repressão da expressão de genes específicos de SM mudou VSMCs para fenótipo proliferativo, promovendo a proliferação e migração de VSMCs e, assim, a formação de neoíntima.^44^

A expressão de lincRNA-p21 foi regulada negativamente nas placas ateroscleróticas. O LincRNA-p21 diminuiu a interação MDM2/p53 e aumentou a interação p300/p53 para facilitar a atividade transcricional de p53, levando à repressão da formação neointimal, a inibição da proliferação celular e o aumento da apoptose em VSMCs e células macrófagos mononucleares in vitro e vivo.^45^

Além disso, alguns outros LncRNAs estão envolvidos no processo AS no nível transcricional, mas as descrições são limitadas. A superexpressão de LncRNA-MeXis em macrófagos pode facilitar a reversão do transporte de colesterol pelos macrófagos através do eixo LXR-MeXis-Abca1, sugerindo que LncRNA-MeXis desempenha um papel protetor no desenvolvimento de aterosclerose.^46^ A expressão ectópica de LncRNA-HOTTIP, induzida por TNF-α ou fator de crescimento derivado de plaquetas (PDGFBB), aumenta a expressão de marcadores proliferativos ciclina D1 e PCNA através da via Wnt/β-catenina, subsequentemente estimulando a proliferação e migração de células endoteliais.^47^ A O-GlcNAcilação modula a ativação do promotor HAS2-AS1, o transcrito anti-senso natural HAS2-AS1 pode regular a transcrição de HAS2 em cis através da remodelação da estrutura da cromatina,^48^ HAS2 pode estar relacionado à proliferação de VSMCs,^49,50^ recrutamento de macrófagos,^50^ migração de VSMCs e formação de neoíntima,^51,52^ e resposta inflamatória.^50,52^ A expressão de LncRNA RP11-714G18.1 na placa aterosclerótica é baixa. Ainda assim, pode regular positivamente a expressão do gene LRP2BP próximo para prejudicar a migração celular, suprimir a adesão de ECs aos monócitos, reduzir a neoangiogênese, diminuir a apoptose de VSMCs e promover a produção de óxido nítrico. Além disso, o LRP2BP sérico foi positivamente relacionado ao colesterol de lipoproteína de alta densidade.^53^

HOXC-AS1 pode suprimir o acúmulo de colesterol em macrófagos por meio da promoção da expressão de HOXC6 em níveis de mRNA.^54^ LEENE pode melhorar a função endotelial aumentando a transcrição inicial do RNA da eNOS.^55^ Lethe Lin et al.,^56^ atua como uma isca lncRNA para interagir com a subunidade RelA do NF-κB e inibe a ligação de RelA ao DNA de genes alvo, como IL6, SOD2, IL8, atenuando a resposta inflamatória.^56^ LncRNA-TSLP induz a transcrição de HOTAIR através da via PI3K/AKT-IRF1, promovendo a proliferação e migração de células endoteliais na aterosclerose.^57^ Além disso, a TSLP induzida por ox-LDL pode se ligar a células dendríticas (DCs) para ativar a inflamação Th^17,58^ que está relacionado à gravidade e progressão da AS.^59^

### Regulação pós-transcricional

Os LncRNAs atuam principalmente como RNAs endógenos competidores (ceRNAs) ou miRNAs “esponja” interagindo com miRNAs no processo de aterosclerose no nível de regulação pós-transcricional. (tabela 1) Além disso, eles também estão envolvidos no controle da tradução, regulação de splicing e o mecanismo de RNA de interferência pequeno (siRNA).^24^

MALAT1 atua como ceRNA na lesão de células induzidas por ox-LDL e desempenha um papel protetor na doença aterosclerose. MALAT1 poderia competir com miR-22-3p por RNA endógeno e regular positivamente os genes alvo CXCR2 e AKT de miR-22-3p para inibir a apoptose de células endoteliais e promover a migração de ECs e angiogênese.60 Cremer S et al.,^61^ descobriram que MALAT1 “esponja” o miR-503 para reduzir a liberação de citocinas pró-inflamatórias, atenuando a inflamação da placa.^61^ Além disso, o supressor de sinalização de citocina 1 (SOCS1) é a proteína alvo do miR-155 que regula negativamente a sinalização do transdutor de sinal da quinase ativada de Janus (JAK) e do ativador da transcrição (STAT). MALAT1 poderia diminuir a regulação de miR-155 e aumentar a expressão de SOCS1 para aliviar a inflamação e apoptose na aterosclerose.^62^ Assim, MALAT1 pode desempenhar um papel protetor por meio da interação com miRNAs na patogênese da aterosclerose.

A expressão de lncRNA H19 foi regulada positivamente em macrófagos tratados com LDL-ox. MiR-130b regula a resposta inflamatória diminuindo os níveis translacionais de TNF-α, Sp1, NF-κB com estimulação lipídica^63^ e inibe a adipogênese ao direcionar PPAR-g.^64^ O silenciamento de H19 aumenta significativamente a expressão de miR-130b, que melhora a inflamação e a síntese de lipídios em células Raw264.7 tratadas com LDL-ox.^65^ O H19 pode acelerar a proliferação e impedir a apoptose em VSMCs estimulados por LDL-ox, suprimindo diretamente a expressão de miR-148b e aumentando a expressão do gene WNT1 do miR-148b alvo.^66^

O LncRNA-MIAT pode estar envolvido na progressão da placa aterosclerótica. O MIAT é expresso principalmente nos macrófagos de placas ateroscleróticas avançadas. Com o tratamento ox-LDL, a expressão de MIAT é regulada positivamente. A molécula anti-fagocítica CD47, um gene alvo de miR-149-5p, está relacionada à depuração de células apoptóticas e núcleos necróticos^67^ O MIAT interfere nas vias do miR-149-5p para aumentar o nível de CD47 em macrófagos, promovendo a vulnerabilidade da placa.^68^ A formação do eixo MIAT/miR-181b/STAT3 desempenha um papel crítico nas células do músculo liso vascular da aorta humana induzidas por ox-LDL (HA-VSMCs) e células mononucleares humanas (U937). O MIAT regula para cima o transdutor de sinal e o ativador do nível da proteína de transcrição 3 (STAT3) por meio do sequestro de miR-181b, promovendo subsequentemente a proliferação, facilitando a parada do ciclo celular e inibindo a apoptose em células HA-VSMCs e U937.^69^

NEAT1 também estava envolvido no processo aterosclerótico como ceRNA, exceto para a remodelação da cromatina no nível transcricional. Wang et al.,^70^ descobriram que NEAT1 foi significativamente regulado positivamente na presença de ox-LDL e serviu como uma esponja para reprimir a expressão de miR-342-3p, aumentando o nível sérico de IL-6, IL-1β, COX-2 e colesterol total levando para acelerar o processo de inflamação e a formação de células espumosas.^70^ LncRNA-TUG1 poderia regular negativamente a expressão de miR-26a e aumentar o mRNA e o nível de proteína de TRPC6 para facilitar a apoptose das células endoteliais.^71^ Zhang et al.,^72^ revelou que o TUG1 esponjou miR-133a e regulou positivamente a expressão do fator de crescimento de fibroblastos 1 (FGF1), resultando em aumento da hiperlipidemia e resposta inflamatória excessiva agravou a lesão aterosclerótica.^72^

Além disso, mais e mais estudos demonstraram que muitos LncRNAs relacionados à aterosclerose desempenham um papel crucial na patogênese da AS, interagindo com miRNAs no nível pós-transcricional. LINC00305 atua como uma esponja endógena para miR-136 e inibe a expressão de miR-136 para suprimir a proliferação de células endoteliais vasculares e aumentar a apoptose.^73^ LincRNA-p21 funciona como ceRNA para promover a apoptose de ECs e induz a progressão do ciclo celular ao direcionar o miR-130b.^74^ LncRNA-GAS5 regula negativamente a expressão de miR-21 para aumentar a expressão de morte celular programada 4 (PDCD4), suprimindo a proliferação de ECs e desencadeando a apoptose de ECs.^75^

### Outras

Os LncRNAs podem funcionar através da localização de proteínas, replicação de telômeros e interferência de RNA em alguns processos,^24^ tal como localização de partículas de RNP em plantas leguminosas, extensão do telômero durante a replicação do DNA em eucariotos,^76^ reduzindo o siRNA gerado por Dicer e afetando a expressão de genes regulados por Dicer.^77^ No entanto seu mecanismo molecular subjacente relacionado ao desenvolvimento de aterosclerose permanece desconhecido.

## Conclusão e Perspectiva

Tomados em conjunto, os LncRNAs podem estar envolvidos em vários processos associados à aterosclerose, incluindo resposta inflamatória, metabolismo lipídico e função celular. Eles regulam a patologia da aterosclerose em níveis epigenéticos, transcricionais e pós-transcricionais, como remodelamento da cromatina, promoção da transcrição e competição endógena por miRNAs. Portanto, os LncRNAs podem servir como novos marcadores diagnósticos e alvos terapêuticos promissores para aterosclerose e doenças vasculares. Além disso, todos esses papéis possíveis nos processos fisiopatológicos abriram espaços para decifrar a função e o mecanismo dos LncRNAs em doenças cardiovasculares e outras doenças, como tumores, doenças renais e nervosas.
