# Supplementary material for: Os LncRNAs Estão Envolvidos no Processo de Aterosclerose em Diversos Níveis
Source: Arq Bras Cardiol. 2022 Jun 6;118(6):1134–40. [Article in Portuguese] doi: 10.36660/abc.20201383 (PMC9345145; doi:10.36660/abc.20201383)
Supplement: Translation

# Translation: LncRNAs are Involved in the Process of Atherosclerosis at Diverse Levels

**Authors:** Shiyi Liang, Weicheng Xv, Chijian Li, Yuxiang Huang, Ge Qian, Yuxiang Yan, Hequn Zou, Yongqiang Li

**Affiliations:** 1 Guangzhou China The Third Affiliated Hospital of Southern Medical University – Department of Nephrology, Guangzhou – China; 2 Beijing China Capital Medical University – Department of Epidemiology and Biostatistics, Beijing – China; 3 Beijing China South China Hospital of Shenzhen University, Department of Nephrology, Beijing – China; 4 Guangzhou China The Third Affiliated Hospital of Southern Medical University – General Practice Department, Guangzhou – Chin

**Keywords:** LnRNAs, Emzymes, Enzyme Inhibitors, Endothelial Cells, Lipoproteins, VLDL, RNA Interference

## Abstract

Atherosclerosis is the most common cause of cardiovascular disease globally, associated with a high incidence of clinical events. Accumulating evidence has elucidated that long non-coding RNAs (lncRNAs) as a novel class of transcripts with critical roles in the pathophysiological processes of atherosclerosis. In this review, we summarize the recent progress of lncRNAs in the development of atherosclerosis. We mainly describe the diverse regulatory mechanisms of lncRNAs at the transcriptional and post-transcriptional levels. This study may provide helpful insights about lncRNAs as therapeutic targets or biomarkers for atherosclerosis treatment.

## Introduction

Cardiovascular diseases (CVDs) are regarded as a global health problem that accounts for 17.9 million deaths every year.^1^ Atherosclerosis (AS), the principal driver of CVDs worldwide, is a lipid-driven chronic inflammatory process with endothelial dysfunction, foam cells formation and final plaque buildup.^2^ This process is accompanied by cells proliferation, apoptosis, and the release of pro-inflammatory factors^3^ (Figure 1). These can trigger plaque rupture and thrombosis formation, leading to acute clinical events, such as stroke and acute coronary syndrome.^4^

**Figure 1:** The pathogenesis of atherosclerosis.

In the mammalian genome, the encoded protein RNAs are only < 3%.^5^ That fraction of the coding gene makes, therefore, hard to explain the complex regulatory mechanism of the organism. In recent years, accumulating studies have revealed the important role of non-coding protein RNAs in the pathophysiological processes of various diseases.^6,7^ According to the length, the non-coding RNAs (ncRNAs) can be divided into long non-coding RNA (lncRNA, >200 nucleotides) and small non-coding RNA (<200 nucleotides, such as miRNAs, piRNAs and siRNAs).^8^ In many researches, some small ncRNAs’ regulatory functions and biological effects have been demonstrated.^9–11^ The function of many lncRNAs is unknown, but an increasing number of lncRNAs have been characterized.

The biosynthesis of lncRNA is similar to that of mRNA. LncRNAs are transcribed by RNA polymerase II but lack open reading frames, and they are in a lower expression than protein-coding genes.^8^ LncRNAs are mainly located within the nucleus and cytoplasm.^12^ In the cytoplasm, lncRNAs can bind with ribosomes^13^ or originate from the mitochondrial genome.^14^ Early reports show that many lncRNAs can’t encode proteins because they lack open reading frames (ORFs) or contain few ORFs. But emerging evidence suggests that some lncRNAs contain small ORFs encoding small proteins or micropeptides, which are regarded as key regulators in various biological processes.^8,15,16^ Studies demonstrate that lncRNAs play critical roles in the function of endothelial and vascular smooth muscle cells (VSMC), macrophage activation, lipid metabolism and inflammatory response.^17,18^ In this review, we mainly discuss the regulation of lncRNAs are involved in the pathophysiologic process of atherosclerosis at transcriptional and post-transcriptional levels.

The pathogenesis of atherosclerosis is accompanied by cell dysfunction, such as proliferation, apoptosis, and migration. The result is foam cells formation and plaque buildup.

### The classifications and regulatory mechanism of LncRNAs

According to the correlation between the genomic location and protein-coding genes, lncRNAs can be divided into (1) intergenic lncRNAs (lincRNAs) that express protein-coding genes as an independent unit. (2) intronic lncRNAs that derive from the introns of protein-coding genes. (3) antisense lncRNAs transcribed from the opposite direction of protein-coding genes. (4) sense lncRNAs that overlap with exons of protein-coding genes on the same strand. (5) enhancers that originate in the enhancer of protein-coding genes. (6) bidirectional lncRNAs that are transcribed from the divergent bidirectional promoters.^19,20^ The criteria of classification also include the various functions in local gene regulation: cis- (regulating proximal genes expression) and trans- (regulating distant genes expression).^21^ Besides, lncRNAs transcripts can also be categorized into linear or circular.^22^

The mechanism of lncRNAs functioning has not been completely elucidated, but it can be classified roughly into several groups: 1. transcriptional regulation is embodied in transcriptional interference, chromatin remodeling and promotion of transcription; 2. post-transcriptional levels manifest in mRNAs splicing regulation translational control and even as sponges for miRNAs; 3. Others contain protein localization, telomere replication, and RNA interference, etc. Furthermore, their targeting mechanisms for regulating gene expression are summarized as the following: signals, decoys, guides and scaffolds.^22,23^

### Transcriptional regulation

LncRNAs can exert their transcriptional regulation through cis-acting and trans-acting mechanisms. (Table 1) LncRNAs regulate neighboring genes expression in cis via transcriptional interference or chromatin remodeling.^24^ Trans-acting lncRNAs can interact with RNA polymerases and transcription elongation factors or serve as a scaffold for chromatin modification complexes to regulate the distant genes.^24,25^

**Table 1:** The role of lncRNAs in the pathologic process of atherosclerosis

	lncRNAs	Mechanism	Effect		References
Cells function			Proliferation	Apoptosis	
Endothelial cells (ECs)	MALAT1	MALAT1-Pc2 (CBX4)-E2F1	+		38
	GAS5	GAS5 - ceRNA (miR-21)	−	+	75
	HOTTIP	TNF-α/PDGFBB-HOTTIP-β-catenin	+		47
	MALAT1	ceRNA (miR-22-3p)		−	60
	TUG1	ceRNA (miR-26a)		+	71
Macrophages, Smooth muscular cells	ANRIL	Bind with CBX7 and SUZ12	+	−	32
	NEAT1	NEAT1-WDR5-SM-specific genes	+		44
	LincRNA-p21	lincRNA-p21-MDM2/ p300-p53	+	−	45
	HAS2	remodeling chromatin structure	+		49,50
	RP11-714G18.1	upregulate LRP2BP expression		−	53
	H19	ceRNA (miR-148b)	+	−	66
	MIAT	ceRNA (miR-181b)	+	−	69

	lncRNAs	Mechanism	Effect	References
Lipid accumulation	MALAT1	MALAT1-CD36-lipid uptake	+	45
	NEAT1	NEAT1-CD36-lipid uptake	−	41
	MeXis	LXR-MeXis-Abca1	−	46
	H19	ceRNA (miR-130b)	−	65
	TUG1	ceRNA (miR-133a)	+	72

	lncRNAs	Mechanism	Effect	References
Inflammatory response	ANRIL	TNF-α/NF-kB-ANRIL/YY1-IL6/8	+	34
	MALAT1	MALAT1-p65/p50-TNF-α and IL-6	−	40
	MALAT1	ceRNA (miR-503 or miR-155)	−	61,62
	H19	ceRNA (miR-130b)	−	
	NEAT1	ceRNA (miR-342-3p)	+	70
	TUG1	ceRNA (miR-133a)	+	72

(+) represents prompt or increase, and

(−) represents prevent or decrease.

The Wellcome Trust Case Control Consortium (WTCCC) study and the genome-wide association studies found that a region on chromosome 9p21 (Chr9p21) was strongly associated with coronary artery disease strongly.^26^ The region is adjacent to a lincRNA named antisense non-coding RNA in the INK4 locus (ANRIL, also known as CDKN2BAS).^27^ Holdt LM et al.^28^ had revealed that ANRIL expression was correlated with atherosclerosis severity by affecting mRNAs’ transcription, and the ANRIL was also detected in atherosclerotic plaques in their study.^28^

Two protein-coding genes, cyclin-dependent kinase inhibitors(CDKN2A, CDKN2B) and the alternative reading frame (ARF) on chromosome 9p21, are tied to ANRIL inextricably, which are tumor suppressors.^27^ The polycomb repressive complex-1 (PRC-1) and polycomb repressive complex-2 (PRC-2) are two kinds of polycomb group proteins involved in maintaining chromatin state.^29^ Their subunits CBX7 and SUZ12 bind ANRIL separately to silence CDKN2A/B locus through H3 lysine27 (K27H3) trimethylation.^30,31^ Yet, the repression of CDKN2A/B may be related to cell proliferation and apoptosis in the atherosclerosis process.^32^

Holdt et al.^28^ found that ANRIL was in a position to exert a regulatory function in distant gene expression in trans. Alu element, marking the promoter of the ANRIL trans-regulated genes, is decisive for linear ANRIL trans-regulation. PcG proteins, triggered by binding with ANRIL, were highly abundant downstream of the Alu motifs.^33^ The recruitment of PcG proteins could regulate the expression of the target genes (TSC22D3, COL3A1) and attenuate ANRIL-mediated pro-atherogenic functions, such as cell adhesion, proliferation, and apoptosis.^3,33^ Furthermore, ANRIL plays a pivotal role in the inflammatory processes through TNF-α/NF-kB-ANRIL/YY1-IL6/8 pathway. PRC-associated proteins Yin Yang 1 (YY1), a transcriptional factor, form a functional complex with ANRIL.^33^ ANRIL-YYI complex binds to IL6/8 promoter loci and stimulates their recruitment in the TNF-α/NF-κB signaling, leading to vascular inflammation.^34^

MALAT1, located on chromosome 11q13, is first described as lncRNA associated with metastasis of lung tumors.^35^ MALAT1 expression is downregulated in atherosclerotic plaques in comparison to non-atherosclerotic arteries.^36^ Michalik et al.^37^ found that silencing of MALAT1 inhibited a switch from a promigratory to a proliferative state of the endothelial cells, resulting in the reduction of vessel growth.^37^ And MALAT1 also acts as a molecular scaffold to interact with unmethylated Polycomb 2 (Pc2); the expression of Pc2 promotes E2F1 SUMOylation and regulates histone modifications to increase cell proliferation.^38^

In a control experiment, Gast et al.^39^ observed that the serum levels of TNF, IL-6, and IFN-γ were increased in the MALAT1-deficient ApoE-/- mice, causing immune dysfunction and aggravated atherosclerosis.^39^ MALAT1 may be involved in the LPS‐induced inflammatory response via LPS/TLR4/NF-κB signaling. MALAT1 interacts with NF-κB subunits p65/p50, inhibiting p65/p50 binding to target promoters such as TNF-α and IL-6, then attenuating an excessive inflammation.^40^

In lipid metabolism, MALAT1 may be upregulated in macrophages during ox-LDL stimulation.^41^ CD36, a class B scavenger receptor, is required for lipid uptake of ox-LDL.^42^ MALAT1 overexpression induces the recruitment of β-catenin on the CD36 promoter to enhance CD36 transcription, promoting lipid uptake in macrophages and accelerating the foam cell formation in atherosclerotic plaques.^41^

NEAT1, an adjacent transcript of MALAT1, can enhance the paraspeckles formation in oxLDL-induced macrophage, which suppresses lipid uptake by binding CD36 mRNA to inhibit CD36 expression and stimulates inflammatory response via phosphorylating p65 to promote TNF-α secretion.^43^ Besides, Ahmed ASI et al.^44^ found that NEAT1 expression was upregulated in vascular smooth muscle cells (VSMCs) after vascular injury in vivo and in vitro, leading to an inactive chromatin state in SM-specific genes through binding with the chromatin modifier WDR5. The repression of SM-specific genes expression switched VSMCs to proliferative phenotype, promoting VSMCs proliferation and migration and thereby neointima formation.^44^

The expression of lincRNA-p21 was downregulated in the atherosclerotic plaques. LincRNA-p21 decreased MDM2/p53 interaction and increased p300/p53 interaction to facilitate the transcriptional activity of p53, leading to the repression of neointimal formation, the inhibition of cell proliferation and the enhancement of apoptosis in VSMCs and mononuclear macrophage cells in vitro and vivo.^45^

Also, some other lncRNAs are involved in the AS process at the transcriptional level, but the descriptions are limited. The overexpression of lncRNA-MeXis in macrophages may facilitate macrophage reversing cholesterol transport via the LXR-MeXis-Abca1 axis, suggesting that lncRNA-MeXis plays a protective role in the development of atherosclerosis.^46^ Ectopic expression of lncRNA-HOTTIP, induced by TNF-α or platelet-derived growth factor (PDGFBB), increases proliferative markers cyclin D1 and PCNA expression through the Wnt/β-catenin pathway, subsequently prompting the endothelial cell proliferation and migration.^47^ The O-GlcNAcylation modulates HAS2-AS1 promoter activation, HAS2-AS1 natural antisense transcript can regulate HAS2 transcription in cis through remodeling chromatin structure,^48^ HAS2 may be related to VSMCs proliferation,^49,50^ macrophages recruitment,^50^ VSMCs migration and neointima formation,^51,52^ inflammatory response.^50,52^ The expression of lncRNA RP11-714G18.1 in atherosclerotic plaque is low. Still, it can upregulate nearby gene LRP2BP expression to impair cell migration, suppress the adhesion of ECs to monocytes, reduce the neoangiogenesis, decrease VSMCs apoptosis and promote nitric oxide production. Furthermore, the serum LRP2BP was positively related to high-density lipoprotein cholesterol.^53^

HOXC-AS1 may suppress the cholesterol accumulation in macrophages via promoting HOXC6 expression at mRNA levels.^54^ LEENE can improve endothelial function by enhancing eNOS initial RNA transcription.^55^ Lethe Lin et al.56 acts as a decoy lncRNA to interact with the NF-κB subunit RelA and inhibits RelA binding to target genes DNA, such as IL6, SOD2, IL8, attenuating the inflammatory response.^56^ LncRNA-TSLP induces HOTAIR transcription through PI3K/AKT-IRF1 pathway, promoting endothelial cell proliferation and migration in atherosclerosis.^57^ Besides, ox-LDL induced TSLP may bind to dendritic cells (DCs) to activate the Th17 inflammation,^58^ which is related to the severity and progression of AS.^59^

### Post-transcriptional regulation

LncRNAs mainly act as competing endogenous RNAs (ceRNAs) or miRNAs “sponge” interacting with miRNAs in the process of atherosclerosis at the post-transcriptional regulation level. (Table 1) Furthermore, they are also involved in translational control, splicing regulation and small interfering RNA (siRNA) mechanism.^24^

MALAT1 acts as ceRNA in ox-LDL-induced cells injury and plays a protective role in atherosclerosis disease. MALAT1 could compete with miR-22-3p for endogenous RNA and upregulate the target genes CXCR2 and AKT of miR-22-3p to inhibit endothelial cells apoptosis and promote the ECs migration and angiogenesis.^60^ Cremer S et al.^61^ found that MALAT1 “sponged” miR-503 to reduce the release of pro-inflammatory cytokines, attenuating plaque inflammation.^61^ Besides, the suppressor of cytokine signaling 1 (SOCS1) is the target protein of miR-155 that negatively regulates Janus activated kinase (JAK)-signal transducer and activator of transcription (STAT) signaling. MALAT1 could downregulate miR-155 and increase the expression of SOCS1 to alleviate the inflammation and apoptosis in atherosclerosis.^62^ Thus, MALAT1 may play a protective role via interacting with miRNAs in the pathogenesis of atherosclerosis.

The expression of lncRNA H19 was up-regulated in ox-LDL treated macrophages. MiR-130b regulates the inflammatory response by decreasing the translational levels of TNF-α, Sp1, NF-κB with lipid stimulation^63^ and inhibits adipogenesis by targeting PPAR-g.^64^ Silencing of H19 significantly increases the expression of miR-130b, which ameliorates inflammation and lipid synthesis in ox-LDL-treated Raw264.7 cells.^65^ H19 can accelerate proliferation and impede apoptosis in ox-LDL-stimulated VSMCs by directly suppressing miR-148b expression and enhancing miR-148b target gene WNT1 expression.^66^

LncRNA-MIAT may be involved in atherosclerotic plaque progression. MIAT is mainly expressed in the macrophages of advanced atherosclerotic plaques. With the ox-LDL treatment, the expression of MIAT is upregulated. Anti-phagocytic molecule CD47, a target gene of miR-149-5p, is related to apoptotic cell clearance and necrotic cores.^67^ MIAT interferes with miR-149-5p pathways to increase the CD47 level in macrophages, promoting plaque vulnerability.^68^ The formation of the MIAT/miR-181b/STAT3 axis plays a critical role in ox-LDL induced human aorta vascular smooth muscle cells (HA-VSMCs) and human mononuclear cells (U937). MIAT up-regulates signal transducer and activator of transcription 3 (STAT3) protein level through sequestering miR-181b, subsequently promoting proliferation, facilitating cell cycle arrest and inhibiting apoptosis in HA-VSMCs and U937 cells.^69^

NEAT1 was also involved in the atherosclerotic process as ceRNA except for remodeling chromatin at the transcriptional level. Lei Wang et al.^70^ found that NEAT1 was significantly upregulated in the presence of ox-LDL and served as a sponge to repress the expression of miR-342-3p, increasing the serum level of IL-6, IL-1β, COX-2, and total cholesterol leading to accelerating inflammation process and the formation of foam cells.^70^ LncRNA-TUG1 could down-regulate the expression of miR-26a and increase the mRNA and protein level of TRPC6 to facilitate the endothelial cells apoptosis.^71^ Lei Zhang et al.^72^ revealed that TUG1 sponged miR-133a and up-regulated fibroblast growth factor 1 (FGF1) expression, resulting in increased hyperlipidemia and excessive inflammatory response aggravated atherosclerotic lesion.^72^

In addition, more and more studies have demonstrated that plenty of atherosclerosis-related lncRNAs plays a crucial role in the pathogenesis of AS by interacting with miRNAs at the post-transcriptional level. LINC00305 acts as an endogenous sponge for miR-136 and inhibits miR-136 expression to suppress the vascular endothelial cells proliferation and enhance apoptosis.^73^ LincRNA-p21 functions as ceRNA to promote ECs apoptosis and induces cell cycle progression by targeting the miR-130b.^74^ LncRNA-GAS5 negatively regulates miR-21 expression to enhance programmed cell death 4 (PDCD4) expression, suppressing ECs proliferation and triggering ECs apoptosis.^75^

### Others

LncRNAs may function through protein localization, telomere replication and RNA interference in some processes,^24^ such as localizing RNP particles in legume plants, extending telomere during DNA replication in eukaryote,^76^ reducing Dicer-generated siRNA and affecting the expression of Dicer-regulated genes.^77^ While their underlying molecular mechanism related to the development of atherosclerosis remains unknown.

## Conclusion and Perspective

Taken together, lncRNAs can be involved in several processes associated with atherosclerosis, including inflammatory response, lipid metabolism and cells function. They regulate the pathology of atherosclerosis at epigenetic, transcriptional and post-transcriptional levels, such as chromatin remodeling, promotion of transcription and competing endogenous for miRNAs. Therefore, lncRNAs may serve as promising novel diagnostic markers and therapeutic targets for atherosclerosis and vascular diseases. Moreover, all of these possible roles in physiopathologic processes have opened venues to decipher the function and mechanism of lncRNAs in cardiovascular diseases and other diseases, such as tumors, renal diseases and nervous diseases.
